# Viral-Induced Rapidly Progressive Alopecia Universalis: A Case Report and Literature Review

**DOI:** 10.7759/cureus.37406

**Published:** 2023-04-10

**Authors:** Manar A Alotaibi, Afrah Altaymani, Abdullah Al-Omair, Waleed Alghamdi

**Affiliations:** 1 Dermatology, College of Medicine, Imam Mohammad Ibn Saud Islamic University, Riyadh, SAU; 2 Dermatology, College of Medicine, Jouf University, Sakaka, SAU; 3 Dermatology, Prince Sultan Military Medical City, Riyadh, SAU; 4 Dermatology, Security Forces Hospital, Riyadh, SAU

**Keywords:** literature review, case report, covid-19, autoimmune disease, alopecia areata

## Abstract

Alopecia areata is a common autoimmune condition that causes a non-scarring form of hair loss. It is associated with several viruses and diseases. One of the viruses that have been linked to alopecia areata is the coronavirus disease of 2019 (COVID-19). It was found to cause the onset, aggravation, or recurrence of alopecia areata in previously infected patients. We report the case of a 20-year-old woman who was medically free and presented with the severe and progressive onset of alopecia areata after one month of contracting COVID-19. This study aimed to explore the literature on COVID-19-associated severe onset alopecia areata in terms of timeline and clinical presentation.

## Introduction

Alopecia areata is a common autoimmune condition that causes a non-scarring form of hair loss. It is generally recognized as a type 1 inflammatory disease. Activated natural killer cell group 2D (NKG2D+) and cytotoxic T cells (CD8+) generate the T-helper cell 1 (Th1) cytokine interferon-gamma (IFN-γ), which disrupts hair follicle immune tolerance and leads to extensive infiltration of inflammatory cells and apoptosis surrounding the hair follicles [1].

Patients with alopecia areata have also been shown to have higher T-helper cell 2 (Th2) cytokine levels, raised blood interleukin-4 (IL-4), interleukin-5 (IL-5), and interleukin-6 (IL-6) levels, high immunoglobulin E (IgE) levels, and elevated eosinophil levels. Scientists have also hypothesized that viral infections may cause alopecia areata [2]. Interferons (IFN) are produced in excess when a virus enters the body. These interferons are one of the elements that disrupt the immune system, resulting in a severe onset of alopecia areata [2,3].

Alopecia areata is associated with several viruses and diseases. Multiple studies have concentrated on and examined the association between alopecia areata and other viruses. They have found that the coronavirus disease of 2019 (COVID-19) can cause the onset, aggravation, or recurrence of alopecia areata in patients who were previously infected with COVID-19 [4].

We present a case report of a 20-year-old woman who is unknown to have any medical illnesses and presented with the severe and progressive onset of alopecia areata after one month of contracting COVID-19. This study aimed to explore the literature on COVID-19-associated severe-onset alopecia areata in terms of timeline and clinical presentation.

## Case presentation

A 20-year-old female patient without a medical history of hair loss arrived at the dermatology department with a sudden onset of diffuse hair loss. Four months ago, when the patient initially presented with anosmia, headache, and sore throat, a polymerase chain reaction confirmed the diagnosis of COVID-19. After one month, she experienced hair loss on her scalp, eyebrows, eyelashes, and the rest of her body. Prior to the infection, neither she nor her family had a history of alopecia areata manifestations.

**Figure 1:**
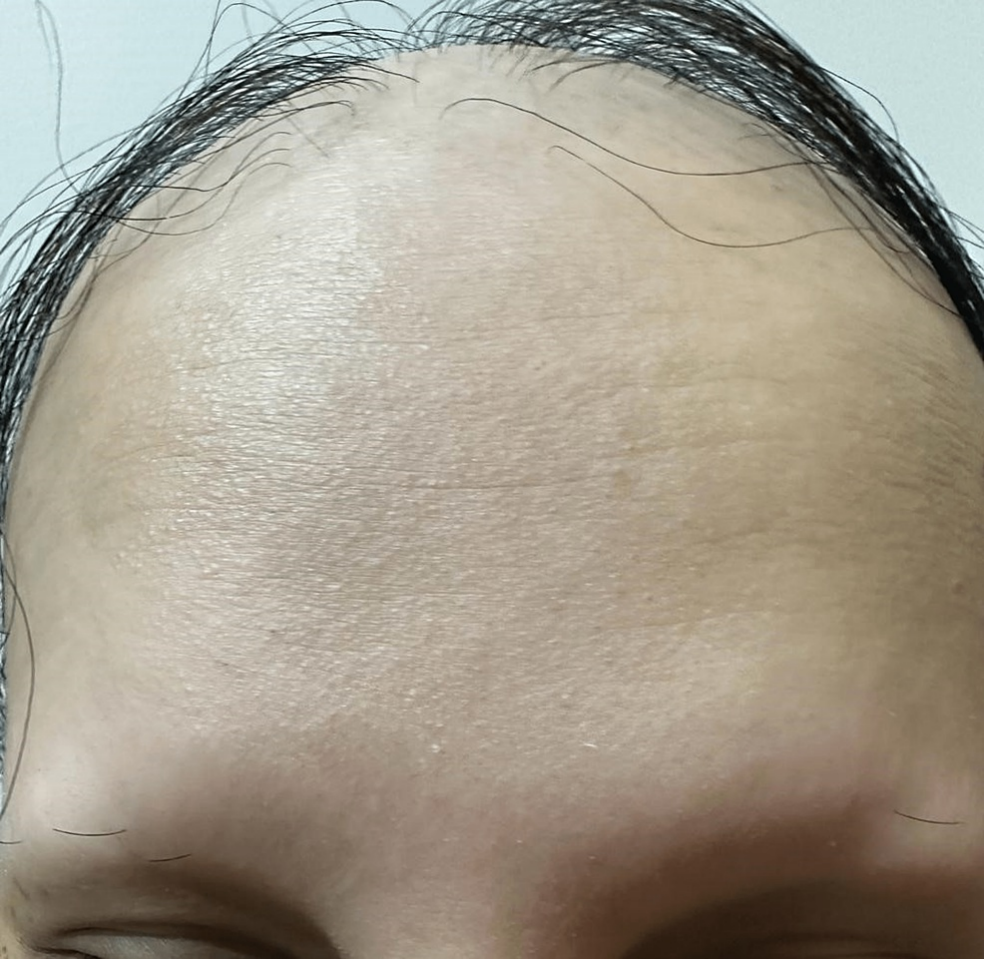
Frontal view of the scalp and eyebrows after one month of COVID-19 infection.

**Figure 2:**
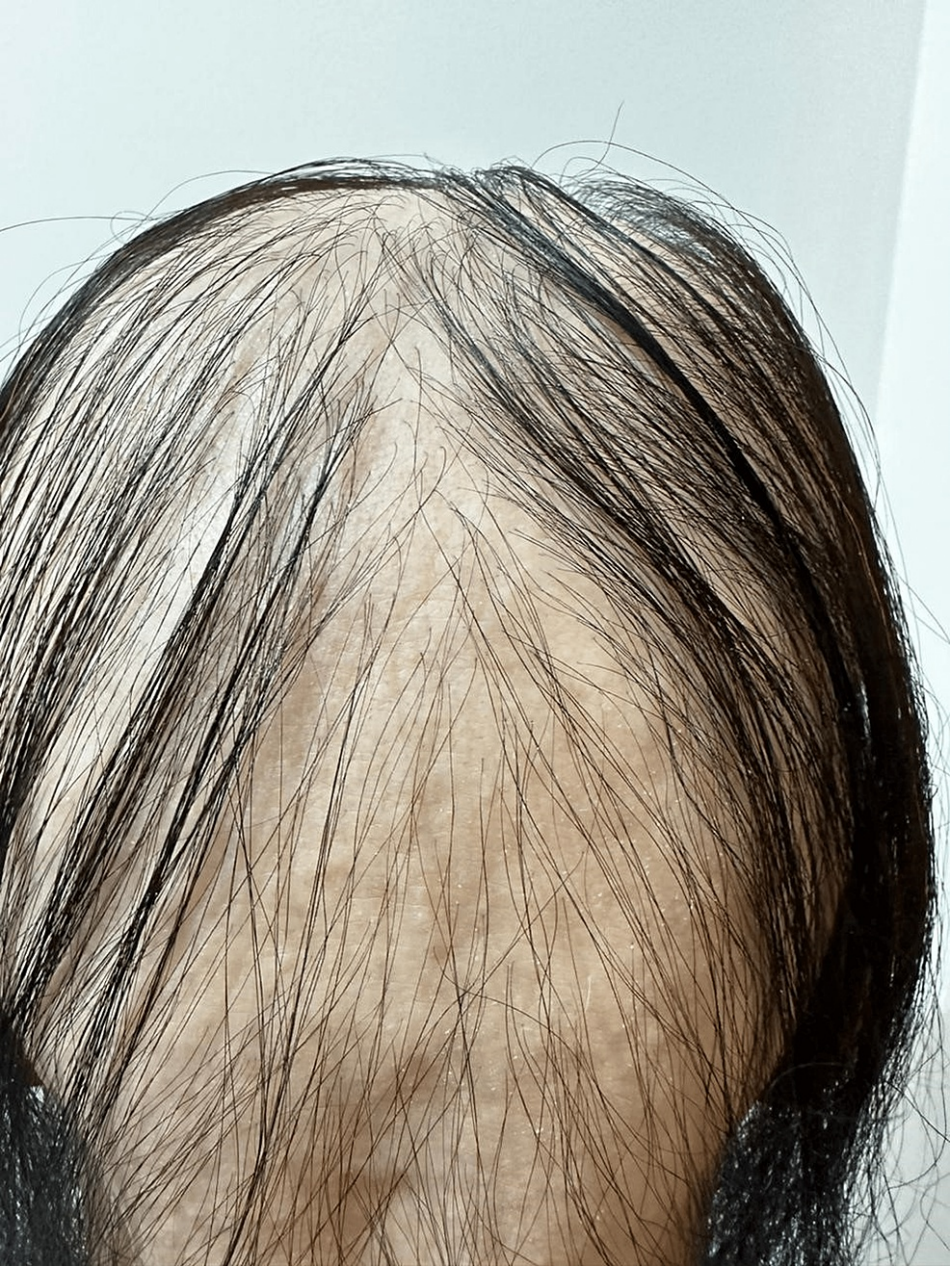
Occipital view of the scalp.

**Figure 3:**
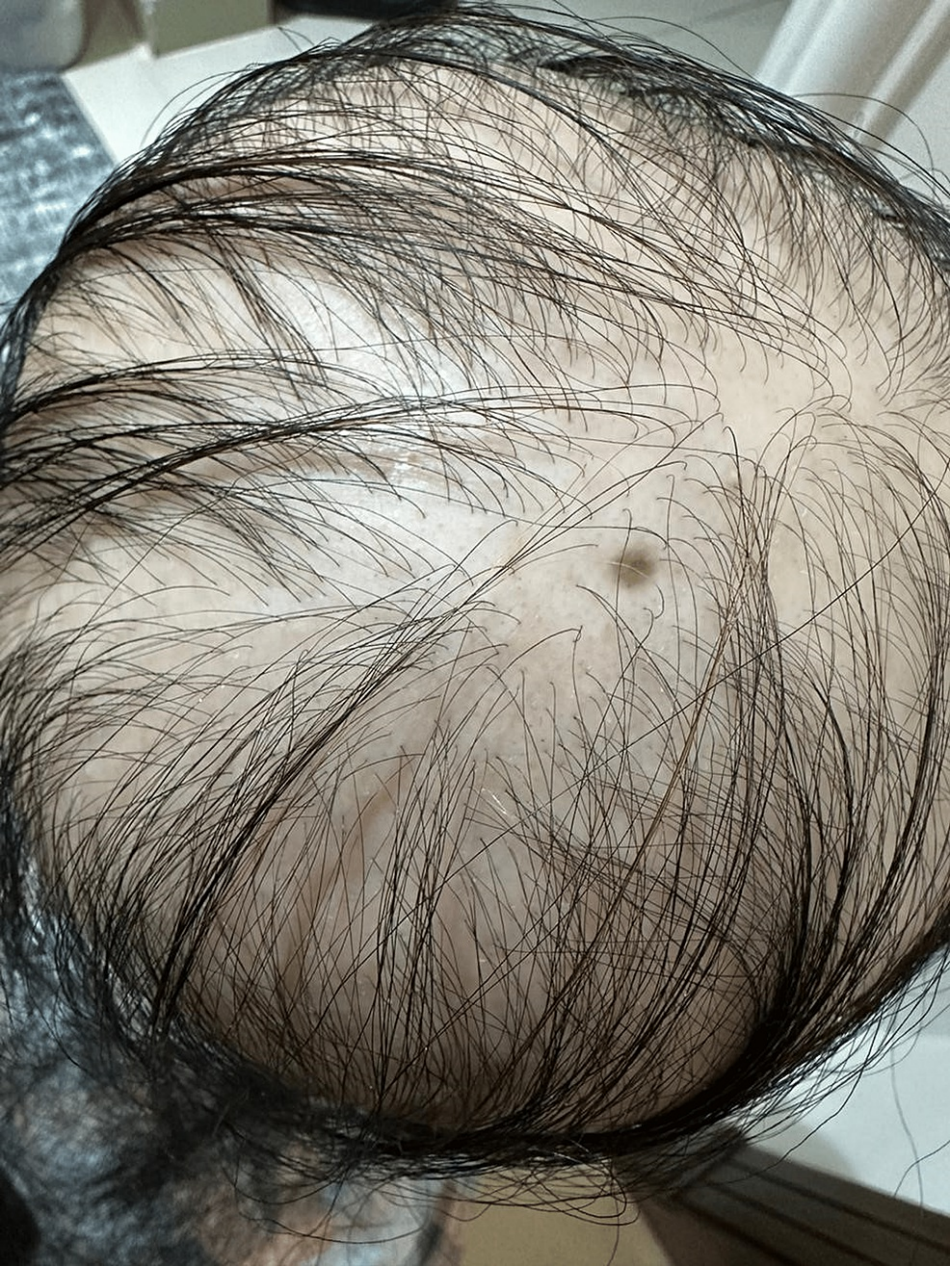
Caudal view of the scalp.

**Figure 4:**
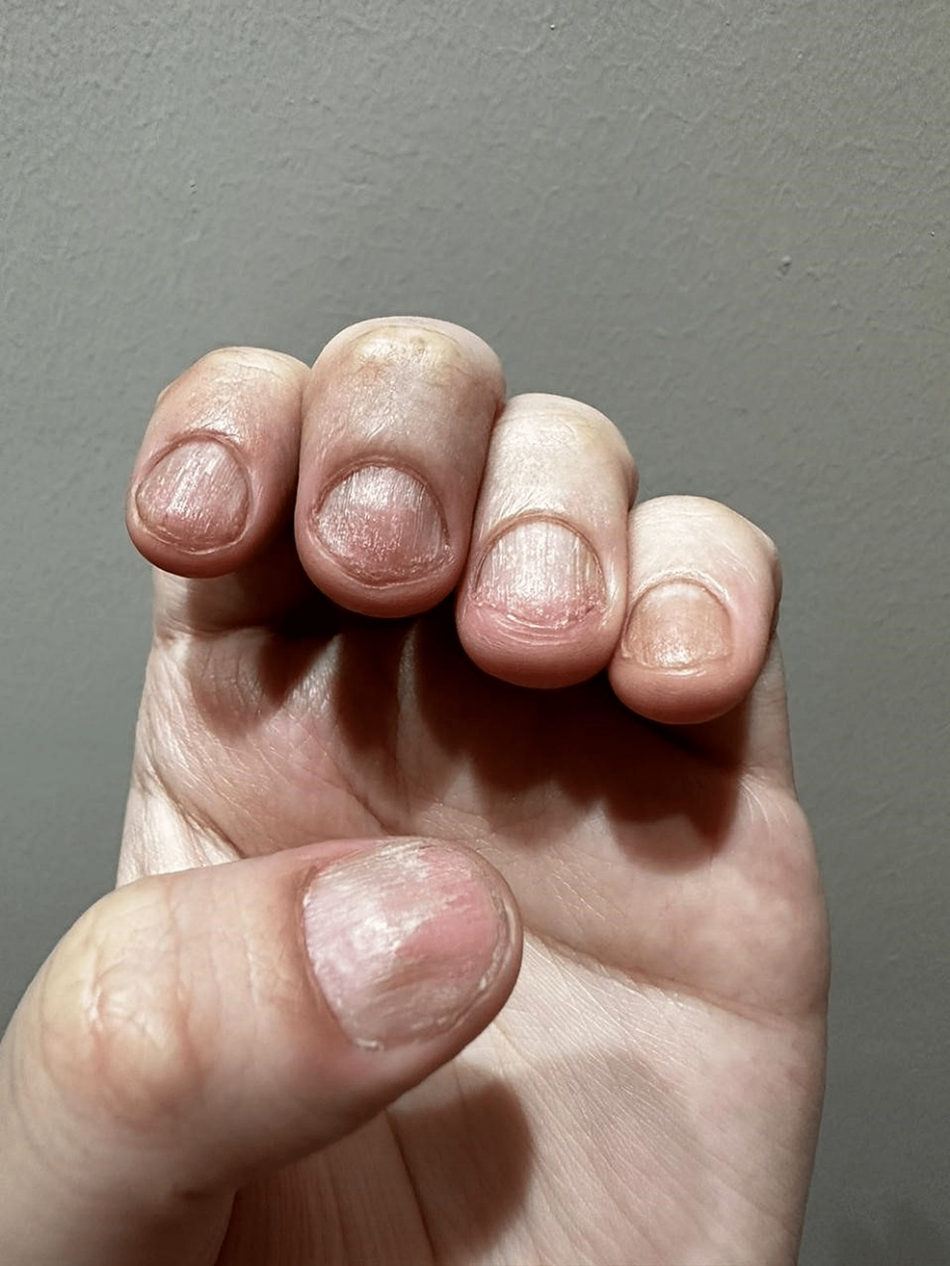
Dystrophic fingernails with regular superficial pitting and nail fragility (trachyonychia).

A clinical examination revealed that her scalp, eyebrows, and the rest of her body were nearly bald. As shown in Figures 1-3, we found dystrophic fingernails with regular superficial pitting and nail fragility (trachyonychia), as shown in Figure 4.

The patient was diagnosed with alopecia universalis based on her typical clinical manifestations and medical history. The Severity of Alopecia Tool (SALT) was then used to assess her hair loss, for which she scored 86.0%, suggesting that her condition was severe.

Laboratory findings were normal, including thyroid function tests, antinuclear antibody (ANA) tests, and an extractable nuclear antigen antibody (ENA) panel. Routine workup, including a complete blood count (CBC), liver function tests (LFT), renal profile, lipid profile, human immunodeficiency virus (HIV) tests, hepatitis B and C serology, and quantiferon gamma tests, were done before starting the patient on the treatment. The results were unremarkable, and the patient started on tofacitinib 5 mg twice daily with an excellent initial response. Table 1 summarizes the literature review on rapid progressive viral-induced alopecia areata.

**Table 1:** Summary of literature on viral induced rapid progressive alopecia areata.

	Study	Years	No of patients	Virus	Alopecia presentation	Severity (SALT score)	Nail involvement	Interval between infection and alopecia
1	Alotaibi et al.	2023	1	SARS‐CoV‐2 (COVID‐19)	Alopecia universalis	SALT score of 86%	Dystrophic fingernails with regular superficial pitting and nail fragility (trachyonychia)	One month after a mild SARS-CoV-2 infection
2	Phong et al. [5]	2022	1	SARS‐CoV‐2 (COVID‐19)	Alopecia universalis	SALT score of 90%	None	One month after a mild SARS-CoV-2 infection
3	Ferreira et al. [6]	2021	3	SARS‐CoV‐2 (COVID‐19)	Focal Alopecia areata and totalis	-	-	-
4	FIvenson [7]	2021	3	SARS‐CoV‐2 (COVID‐19)	Focal alopecia areata and totalis	One case present with Hair loss >99% of all body hair	Non mention	One case after two months of infection
5	Rossi et al. [8]	2021	1	SARS‐CoV‐2 (COVID‐19)	Alopecia totalis	-	Non mention	One month after a mild SARS-CoV-2 infection
6	Ito et al. [9]	2013	1	Human T-cell lymphotropic virus-1-associated myelopathy	Alopecia totalis	Non mention	Non mention	-
7	Rodriguez et al. [10]	2008	12	Epstein-barr virus infection	3 out of 12 had alopecia universalis	Non mention	Non mention	Alopecia onset was within 1 week up to 6 months after infectious mononucleosis infection

## Discussion

This study aims to present the available literature on viral-induced alopecia areata. In our study, a female patient was diagnosed with severe alopecia universalis one month after her recovery from the COVID-19 infection. This confirmed the hypothesis demonstrated in previous literature that viral infections are a risk factor for its development. COVID-19 could disrupt the immunological privilege of the hair follicle by activating CD8+ cytotoxic cells and increasing the release of IFN gamma, resulting in a massive immune response and cell destruction [4].

Phong et al. present a case similar to our finding in a 28-year-old female with rapid diffuse alopecia universalis one month after a mild SARS-CoV-2 infection [5]. Confirming the findings of our case report, a previous case report in Brazil reported a case of a 24-year-old female with alopecia areata totalis who tested positive for COVID-19 while taking tofacitinib 5 mg twice a day (BID) throughout the past year [6].

Another study in 2020 by FIvenson reported three case reports. One person developed complete hair loss after recovery from COVID-19 infection after one month. This confirms the timeline of our result, as it has been documented in other studies [7,8]. Another study documented a case report for a 38-year-old female diagnosed with total alopecia areata and human T-cell lymphotropic virus-1-associated myelopathy (HAM). In these alopecia totalis patients, autoreactive and cytotoxic CD8(+) T lymphocytes generate alopecia areata and HAM, as suggested by this observation [9]. The connection between viral infection and IFN therapy with alopecia areata was verified by a study by Richardson et al. It demonstrates that the hepatitis B surface antigen protein shares epitopes with human killer immunoglobulin-like receptors [11].

Unexpectedly, in 2022, Chidiac et al. presented a case in Lebanon for a 32-year-old female who had alopecia totalis for 13 years, which resolved spontaneously after a SARS-CoV-2 infection [12].

COVID-19 and other viruses can significantly trigger or induce remission of alopecia areata with different presentations. Further studies are needed to educate on the possible pathogenesis and help predict the outcomes of the disease.

## Conclusions

Our literature review and case report confirm the findings of the available literature on the association between viral infections and the development of alopecia-related hair loss. This phenomenon could be justified by the immune response to the viral infection that might trigger the development of the disease.

## References

[1] Ito T, Kageyama R, Nakazawa S, Honda T (2020). Understanding the significance of cytokines and chemokines in the pathogenesis of alopecia areata. Exp Dermatol.

[2] Rajabi F, Drake LA, Senna MM, Rezaei N (2018). Alopecia areata: a review of disease pathogenesis. Br J Dermatol.

[3] Meah N, Wall D, York K (2021). The Alopecia Areata Consensus of Experts (ACE) study part II: results of an international expert opinion on diagnosis and laboratory evaluation for alopecia areata. J Am Acad Dermatol.

[4] Christensen RE, Jafferany M (2022). Association between alopecia areata and COVID-19: a systematic review. JAAD Int.

[5] Phong CH, Babadjouni A, Nguyen C, Kraus CN, Mesinkovska NA (2022). Not just thinning: a case of alopecia universalis after mild COVID-19. JAAD Case Rep.

[6] Berbert Ferreira S, Gavazzoni Dias MF, Berbert Ferreira R, Neves Neto AC, Trüeb RM, Lupi O (2021). Rapidly progressive alopecia areata totalis in a COVID-19 patient, unresponsive to tofacitinib. J Eur Acad Dermatol Venereol.

[7] FIvenson D (2021). COVID-19: association with rapidly progressive forms of alopecia areata. Int J Dermatol.

[8] Rossi A, Magri F, Michelini S (2021). New onset of alopecia areata in a patient with SARS-CoV-2 infection: Possible pathogenetic correlations?. J Cosmet Dermatol.

[9] Ito T, Shimada S, Mori T, Tokura Y (2013). Alopecia areata possibly induced by autoimmune reaction in a patient with human T-cell lymphotropic virus-1-associated myelopathy. J Dermatol.

[10] Rodriguez TA, Duvic M (2008). Onset of alopecia areata after Epstein-Barr virus infectious mononucleosis. J Am Acad Dermatol.

[11] Richardson CT, Hayden MS, Gilmore ES, Poligone B (2018). Evaluation of the relationship between alopecia areata and viral antigen exposure. Am J Clin Dermatol.

[12] Chidiac G, Chrabieh R, Maamari M, El Khoury J, Ayoub N (2022). Spontaneous complete resolution of alopecia totalis post SARS-CoV-2 infection. JAAD Case Rep.

